# Multitask fMRI and machine learning approach improve prediction of differential brain activity pattern in patients with insomnia disorder

**DOI:** 10.1038/s41598-021-88845-w

**Published:** 2021-04-30

**Authors:** Mi Hyun Lee, Nambeom Kim, Jaeeun Yoo, Hang-Keun Kim, Young-Don Son, Young-Bo Kim, Seong Min Oh, Soohyun Kim, Hayoung Lee, Jeong Eun Jeon, Yu Jin Lee

**Affiliations:** 1 Department of Psychiatry and Center for Sleep and Chronobiology, Seoul National University College of Medicine, Seoul, Republic of Korea; 2 Department of Biomedical Engineering Research Center, Gachon University, Inchon, Republic of Korea; 3 Department of Biomedical Engineering, Gachon University, Inchon, Republic of Korea; 4 Department of Neurosurgery, Gachon University Gil Hospital, Inchon, Republic of Korea; 5 Department of Psychiatry, Dongguk University Hospital, Ilsan, Republic of Korea; 6 Department of Neurology, Gangneung Asan Hospital, Gangneung, Republic of Korea

**Keywords:** Circadian rhythms and sleep, Psychiatric disorders

## Abstract

We investigated the differential spatial covariance pattern of blood oxygen level-dependent (BOLD) responses to single-task and multitask functional magnetic resonance imaging (fMRI) between patients with psychophysiological insomnia (PI) and healthy controls (HCs), and evaluated features generated by principal component analysis (PCA) for discrimination of PI from HC, compared to features generated from BOLD responses to single-task fMRI using machine learning methods. In 19 patients with PI and 21 HCs, the mean beta value for each region of interest (ROIbval) was calculated with three contrast images (i.e., sleep-related picture, sleep-related sound, and Stroop stimuli). We performed discrimination analysis and compared with features generated from BOLD responses to single-task fMRI. We applied support vector machine analysis with a least absolute shrinkage and selection operator to evaluate five performance metrics: accuracy, recall, precision, specificity, and F2. Principal component features showed the best classification performance in all aspects of metrics compared to BOLD response to single-task fMRI. Bilateral inferior frontal gyrus (orbital), right calcarine cortex, right lingual gyrus, left inferior occipital gyrus, and left inferior temporal gyrus were identified as the most salient areas by feature selection. Our approach showed better performance in discriminating patients with PI from HCs, compared to single-task fMRI.

## Introduction

Insomnia is a common, distressing, and clinically important symptom, which causes difficulty initiating sleep; frequent awakening; or early-morning awakening with daytime dysfunction. Approximately one-third of the general population experiences insomnia symptoms throughout their lifetime^1^; 1-year follow-up of patients with insomnia revealed that 69% exhibited chronic symptoms^2^. Because of its high prevalence and frequent associations with medical and psychiatric disorders^3,4^, there is a need to study the neurobiological mechanisms of insomnia to improve quality of life and reduce socioeconomic burden.

According to the International Classification of Sleep Disorders Second Edition (ICSD-2), psychophysiological insomnia (PI) is a form of insomnia that comprises heightened arousal and learned sleep-preventing associations, which cause insomnia and poor functioning during wakefulness^5^. Patients with PI frequently complain of sleep-related worries and ruminations that may reduce cognitive performance^6^.

Hyperarousal is an important model to understand the pathophysiology of insomnia. In terms of sleep–wake regulation neurobiology, insomnia is presumed to result from dysregulation of arousal networks including the hypothalamus, brain stem, and cortical areas^7–9^. Numerous physiological studies in patients with insomnia have supported hyperarousal models. Patients with insomnia had significantly higher heart rates in sleep and wake states than those of control groups^10,11^. Moreover, a cortical arousal study indicated that beta electroencephalogram frequencies increased during non-rapid eye movement sleep in adolescent patients with insomnia^12^. Notably, patients with hyperarousal insomnia show inhibited automaticity to sleep by selective attention (attentional bias) to sleep-related stimuli, and insomnia symptoms become chronic via sleep-related maladaptive behavior^8^. Espie et al. suggested that the attention system of patients with insomnia may be sensitive to sleep-related stimuli, thus constituting an important cognitive factor that influences the development and maintenance of insomnia^13^. Previous neurophysiological research indicated that patients with insomnia typically demonstrate cognitive hyperarousal via differences in vigilance to sleep-related stimuli^14,15^. Studies using an emotional Stroop task showed that patients with PI responded more slowly to sleep-related words, compared with control individuals^16^. Other studies supported attentional salience in patients with PI, based on comparisons of sleep-related and control objects^17,18^.

While psychophysiological experiments related to hyperarousal insomnia have been actively investigated, there have been few task-related neuroimaging studies involving the provision of sleep-related stimuli for evaluation of hyperarousal reactions in patients with PI. Thus far, the results of these studies have been inconsistent. Baglioni et al. reported that, compared with good sleepers, patients with insomnia disorder presented heightened activity in the amygdala in response to insomnia-related pictures^19^. In a previous study of 14 patients with PI, the regional brain activity in response to sleep-related sound was reportedly reduced in the left middle temporal and left middle occipital gyri after cognitive behavioral therapy for insomnia^20^. Furthermore, blood oxygen level-dependent (BOLD) signals in response to sleep-related pictures were significantly higher in the PI group than in good sleepers in the bilateral precentral, left prefrontal, left fusiform, and bilateral posterior cingulate cortices^21^. Hwang et al. reported that clinical improvement of insomnia symptoms after insomnia cognitive–behavioral therapy was correlated with changes in Stroop task-related brain activities associated with cognitive processes in patients with PI, although there were no differences in brain activation related to the Stroop task between patients with PI and good sleepers^22^. Furthermore, Spiegelhalder et al. suggested that the use of sleep-related pictorial stimuli is needed for investigation of sleep-related cognitive processes because patients with insomnia did not significantly differ from control individuals in terms of brain activity, evaluated by an emotional Stroop task using sleep-related words and neutral words^23^.

The use of task neuroimaging techniques allows for detect changes in brain activity related to cognitive dysfunction and compensatory response in insomnia patients^24^. The Stroop task is a color-naming task for evaluation of executive function, which might show subjective or objective deterioration in patients with insomnia^25^. Previous neuroimaging studies that evaluated cognitive dysfunction in patients with insomnia were limited in that they used mainly cognitive tasks, which evaluate working memory^26–30^. As mentioned earlier, the results of single task-based functional magnetic resonance imaging (fMRI) studies in insomnia were inconsistent and limited. In current study, it was hypothesized that multi-task fMRI method would be meaningful for understand hyperarousal network and dysfunctional cognitive processes in insomnia patients. fMRI was used to identify specific cognitive process evoked by a specific task linked to insomnia. Analyzing each task separately shows differential activations but cannot capture shared information between tasks and voxels which might be crucial to specify neural representation of insomnia. In this respect, it needs for a data-driven approach to properly describe the spatial distribution of neural representation and to extract the relevant features from multi-task fMRI results on insomnia^31,32^. Motivated by the success of machine learning in terms of feature reduction and image classification, this study proposes a new framework to improve the detection of differential brain activity in patients with insomnia disorder by machine learning, based on neural responses to multitask fMRI. fMRI yields stimulus-induced images in a participant during different tasks and allows the extraction of potential commonalities among tasks^33^. In the present study, we applied principal component analysis (PCA) and support vector machine (SVM) analysis with a least absolute shrinkage and selection operator (LASSO) to capture voxel covariance patterns of BOLD responses to multitask fMRI and to construct a cross-validated model for distinguishing patients with PI from healthy controls (HCs). PCA is an unsupervised multivariate technique that transforms the original variables into new variables, such that the original variables are projected onto principal components (PCs), based on the covariance of the original variables. Loading values in PCs, with respect to fMRI, represent positive or negative correlations among cortical areas. In previous neuroimaging studies, PCA was used successfully to describe the biological processes of disease-relevant spatial patterns^34,35^ and to extract relevant features in neuroimaging classification^36,37^. PCA with multitask fMRI may aid in the exploration of hyperarousal and dysfunctional cognitive processes in multiple domains of the brain in patients with insomnia.

We previously published the results for each single task^20–22^. Here, we analyzed the differences for each task and compared patients with PI to HCs, by means of multiple tasks by PCA. The present study was performed: (1) to investigate how the spatial covariance pattern of BOLD responses to sleep-related multitask fMRI differs between patients with PI and HCs, and (2) to construct features optimized for differentiation of patients with PI from HCs by machine learning. To address these aims, we first examined and compared the spatial covariance patterns of patients with PI and HCs captured by PCA. We then selected several PCs using LASSO as input features for the SVM. Finally, we compared five performance metrics—accuracy, recall, precision, specificity, and F2—between the features from PCA and BOLD responses to three single-task fMRI.

## Results

### Demographic, clinical, and polysomnographic variables of all study participants

Table 1 summarizes the clinical and polysomnographic characteristics of all study participants. As shown in Table 1, there were no significant differences in age (*p* = 0.409, *t* test) or sex (*p* = 0.807, chi-squared test) between patients with PI and HCs. However, patients with PI had higher Pittsburgh Sleep Quality Index, Epworth Sleepiness Scale, and Beck Anxiety Inventory scores, compared with HCs (all *p* < 0.05, *t* test). The time in bed, total sleep time, and sleep efficiency derived from nocturnal polysomnography were significantly lower in patients with PI than in HCs. The maximum heart rate and spontaneous arousal index of patients with PI were significantly higher than those of the HCs (*p* < 0.05, Mann–Whitney test), while the minimum and average heart rates were higher in the PI group, but this difference was not statistically significant.

**Table 1:** Demographic, clinical, and polysomnographic variables of all study participants.

	PI (*n* = 19)	HC (*n* = 21)	*P* value
Age (years)	46.78 ± 12.36	39.71 ± 13.34	0.409
Sex (M/F)	4/15	4/17	0.807
PSQI	13.39 ± 3.81	4.67 ± 2.33	**0.003**
ESS	9.67 ± 5.85	6.29 ± 3.32	**0.024**
DBAS	97.56 ± 18.22	64.71 ± 24.39	0.529
BDI	10.33 ± 8.57	6.62 ± 6.93	0.270
BAI	9.94 ± 7.70	4.19 ± 3.79	**0.041**
**Nocturnal PSG**			
TIB (min)	431.47 ± 83.96	467.73 ± 24.03	**0.016**
TST (min)	375.67 ± 89.14	430.80 ± 38.42	**0.012**
SE (%)	82.15 ± 11.50	89.18 ± 8.02	**0.032**
SL (min)	14.61 ± 16.46	13.73 ± 10.17	0.126
WASO (min)	62.53 ± 38.83	39.13 ± 37.96	0.334
N1 (%)	15.16 ± 7.77	11.20 ± 5.92	0.353
N2 (%)	60.21 ± 7.52	60.10 ± 6.02	0.221
N3 (%)	5.36 ± 5.61	7.47 ± 5.70	0.959
REM sleep (%)	19.26 ± 7.28	20.88 ± 5.23	0.452
AHI	3.50 ± 4.44	2.49 ± 2.86	0.121
PLMI	3.33 ± 4.69	3.57 ± 12.16	0.448
Maximum heart rate	112.9 ± 19.19	97.1 ± 11.17	0.020
Minimum heart rate	54.6 ± 3.51	53.9 ± 5.42	0.851
Average heart rate	63.1 ± 6.81	60.1 ± 7.93	0.059
Spontaneous arousal index(cortical arousal per hour)	7.4 ± 7.05	4.3 ± 5.76	0.041

Clinical data: nPSG data, PI group *n* = 18; nPSG data, HC group *n* = 20.

*BDI *Beck Depression Inventory, *DBAS* Dysfunctional Beliefs and Attitudes about Sleep Scale, *ISI* Insomnia Severity Index, *N1* stage 1, *N2* stage 2, *N3* stage 3, *PSG* polysomnography, *PSQI* Pittsburgh Sleep Quality Index, *REM* rapid eye movement, *SE* sleep efficiency, *SL* sleep latency, *TIB* time in bed, *TST* total sleep time, *WASO* waking time after sleep onset, *AHI* Apnea–Hypopnea Index.

### Visual analysis of cortical BOLD activation for picture, sound, and Stroop stimuli

**Figure 1:**
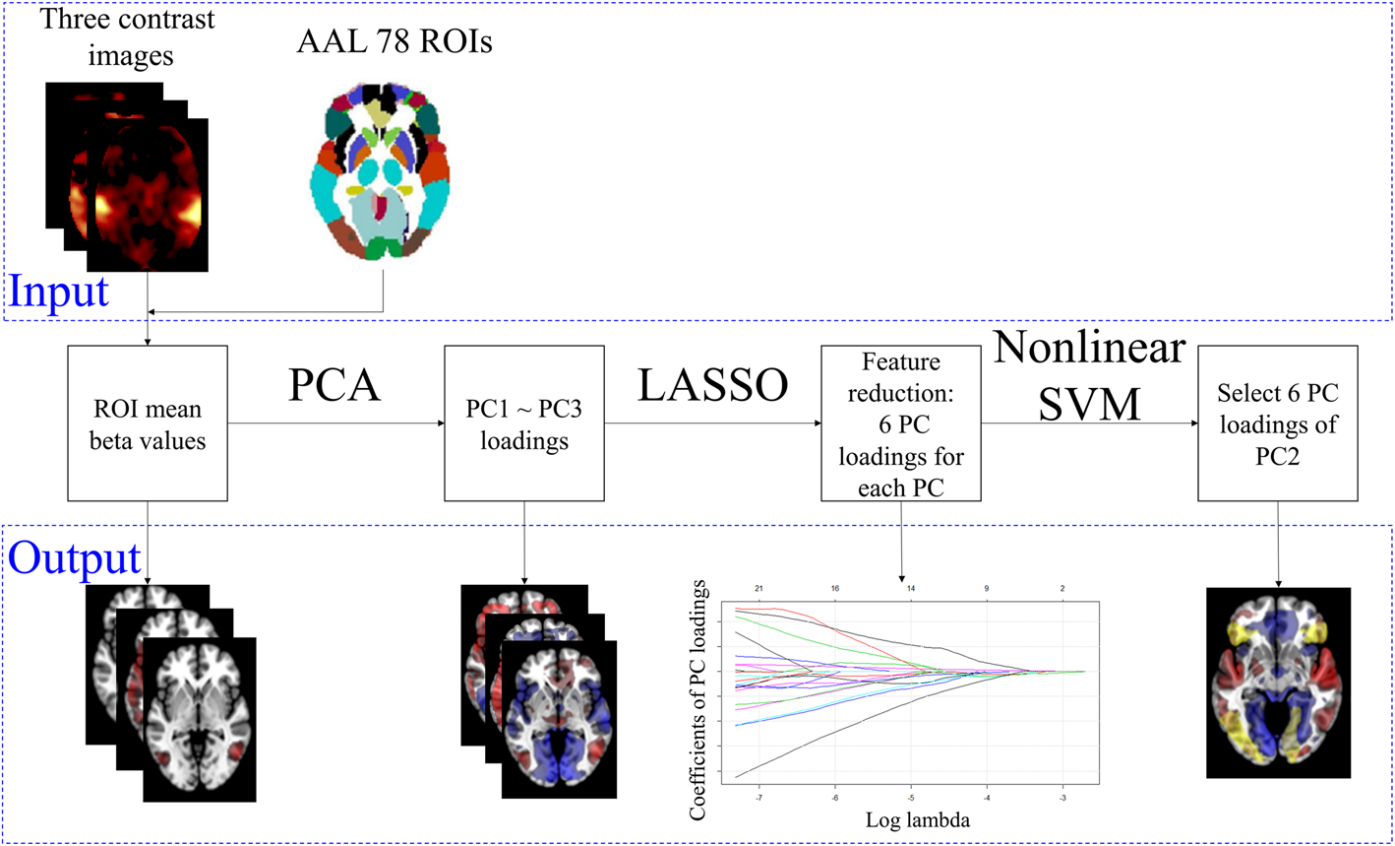
Analytical procedure of the machine learning approach based on multitask fMRI data. The proposed machine learning approach based on multitask fMRI data is shown. The upper box shows input data, and the lower box shows output data. Output data are displayed as voxel-based results to clarify the overall procedure.

**Figure 2:**
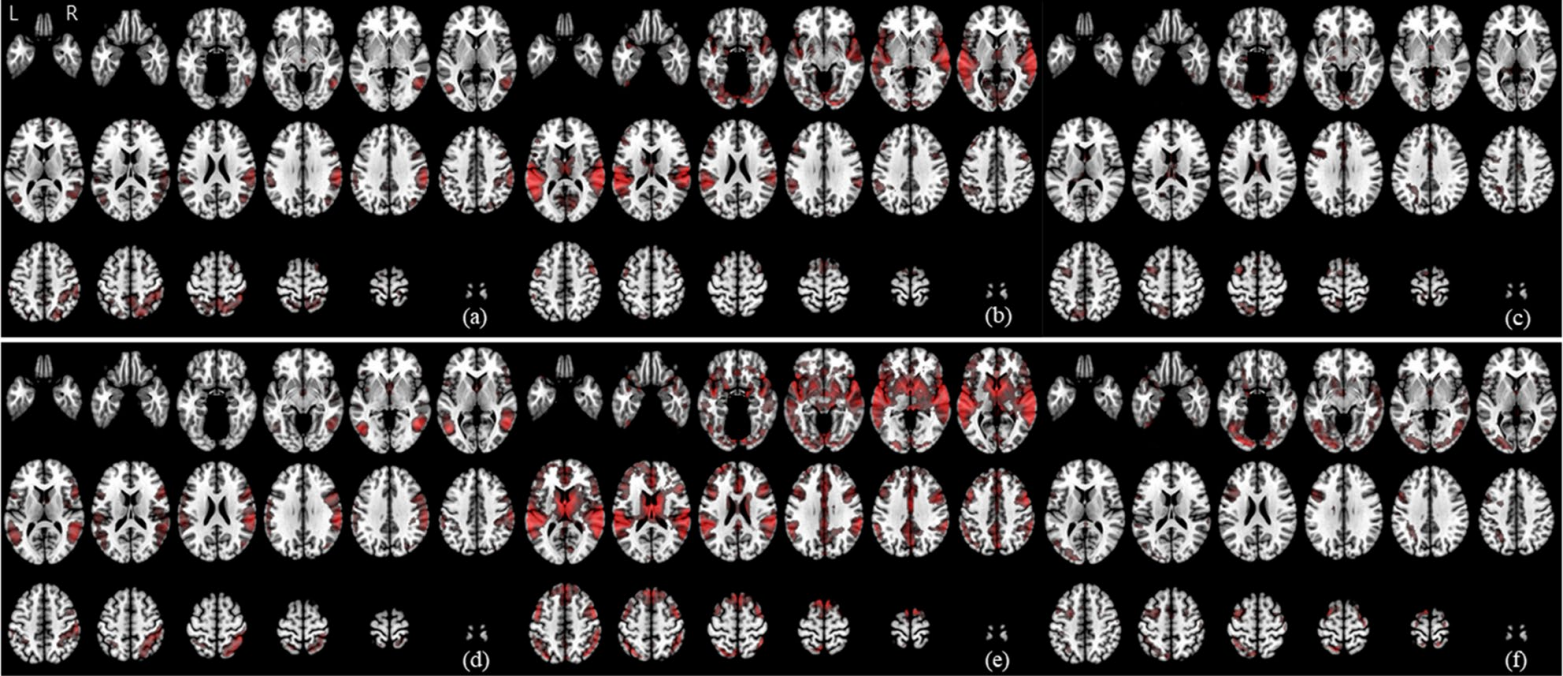
Representative mean cortical BOLD signal amplitudes for picture, sound, and Stroop stimuli. The mean beta values for picture (**a**), sound (**b**), and Stroop (**c**) stimuli in the PI group and for picture (**d**), sound (**e**), and Stroop (**f**) stimuli in the HC group (color scale = 0.1–0.5) are shown. The color scale represents the height of the BOLD signal (beta value) in the task (picture = sleep-related—neutral, sound = sleep-related—white noise, and Stroop = incongruent—congruent).

Figure 1 shows the flow of machine learning based on multitask fMRI data. To depict the BOLD responses for picture, sound, and Stroop stimuli, mean contrast images for each group were overlaid on the T1-weighted template image. Sleep-related picture stimuli elicited increased BOLD signals in both the temporoparietal junction and posterior parietal cortex (right hemisphere dominant) in both patients with PI and HCs (Fig. 2). Sleep-related sound stimuli elicited increased BOLD signals mainly in both the temporal and insular cortexes, including the medial region of the thalamus, in both patients with PI and HCs. The entire thalamus, caudate, inferolateral and medial prefrontal and parietal cortexes, as well as relatively larger involvements in the temporal cortex, were also observed in HCs. For word Stroop stimuli, the dorsolateral prefrontal and parietal (left dominant) cortexes exhibited increased BOLD signals in both patients with PI and HCs. The fusiform and occipital cortexes showed larger involvements in HCs than in patients with PI during exposure to Stroop stimuli. The voxel-wise approach has a large number of features and high computational costs; thus, the region of interest (ROI) approach was used to generate input features. The main benefits of the ROI approach are an increased signal-to-noise ratio with reasonable feature dimensions. Estimated median and range of mean beta values for each ROI (ROIbvals) are shown in Supplementary Fig. S1.

### Feature selection with PC loadings

**Figure 3:**
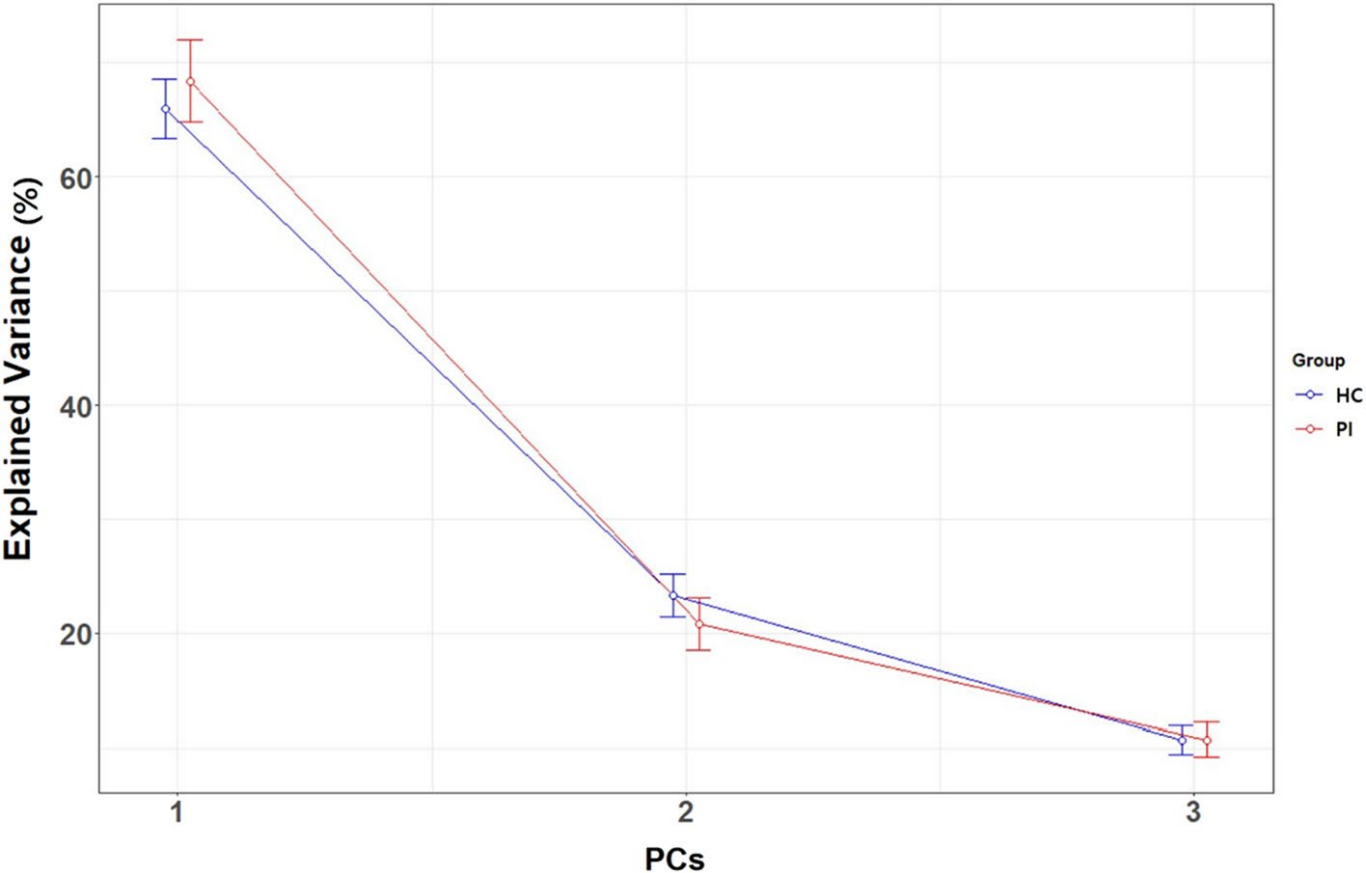
Principal component analysis showing explained variance proportions of the three PCs for ROIbvals of the three contrast images. PCs on the x-axis are ranked in descending order according to the proportions of explained variance of PCs for ROIbvals of the three contrast images (i.e., picture, sound, and Stroop) in the PI (red) and HC (blue) groups. PC1, PC2, and PC3 explain approximately 70%, 20%, and 10% of the total variance of the raw data, respectively.

**Figure 4:**
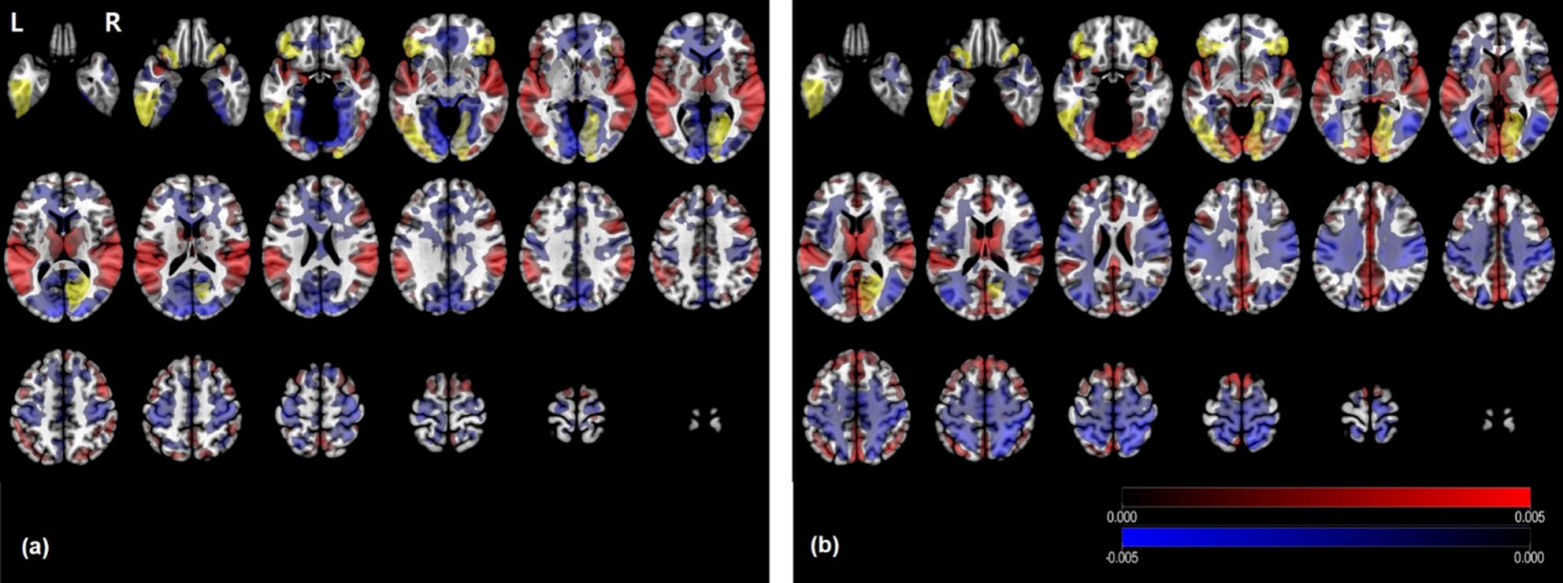
Representative PC2 images of participants in the PI and HC groups. Voxel-based PC2 images were generated using PC2 loadings resulting from mean contrast images of picture, sound, and Stroop stimuli for participants in the PI (**a**) and HC (**b**) groups. Red represents positive loadings and blue negative loadings, indicating that neuronal activation was inversely correlated in these structures. Yellow represents selected ROI-based features by LASSO. Spatial pattern showed the most significant overlap in sound BOLD activation.

Figure 3 and Table 2 show the explained variance proportion of the three PCs resulting from Eq. (1). The first PC explained approximately 70% of the variance (PI, 68.40 ± 3.62; HC, 66.02 ± 2.61; mean ± SEM) and the second PC explained approximately 20% of the variance (PI, 20.84 ± 2.32; HC, 23.31 ± 1.84). The remaining component explained the remaining 10% of the variance (PI, 10.76 ± 1.51; HC, 10.67 ± 1.25). In ROI-based feature selection with a PC component using Eq. (2), only involving the PC2 component, six features were selected as the effective features for differentiation between patients with PI and HCs at the point where minimum deviance was obtained ($\uplambda =0.0122$; Supplementary Table S1). The areas included the bilateral inferior frontal gyrus (orbital), right calcarine cortex, right lingual gyrus, left inferior occipital, and left inferior temporal gyrus (shown in yellow in Fig. 4). There were no effective features in PC1 and PC3 at the minimum deviance criterion. To determine whether the PC loading approach could be a better predictor, compared with BOLD signal change for individual stimuli, we also chose six features from ROIbvals of each of the picture, sound, and Stroop runs. Supplementary Table S1 shows selected features resulting from feature selection with PC2 and ROIbvals of picture, sound, and Stroop runs. For the purpose of PC2 interpretation, voxel-based PC images (PC loadings) were generated using mean contrast images of each group. Voxel-based PC2 images, which showed the overlap with sound activation, demonstrated high positive loading in the bilateral temporal cortex, thalamus, and parietal cortex in patients with PI; they showed high negative loading in the orbitofrontal cortex and lingual gyrus (Fig. 4). These observations indicated that neuronal activation was inversely correlated in these structures. Notably, in HCs, high positive loading was observed in the mediofrontal and lingual gyri, where negative loading was observed in patients with PI, as well as in bilateral temporal cortex and thalamus. In further analysis of patients with PI, PC1 had high positive loading in both the parietal (right hemisphere dominant) and medial frontal cortex, whereas it had high negative loading in both the temporal and lingual gyri. With regard to PC3, high positive loading was observed in the inferolateral prefrontal cortex, insular cortex, and the heads of the caudate nuclei; high negative loading was observed in the high parietal cortex (Supplementary Fig. S2). In comparison with the BOLD signal pattern in patients with PI, the PC1 pattern was the most closely related to picture activation and PC3 to Stroop activation.

**Table 2:** Summary statistics of explained variance proportion of the three principal components.

Group	PC	Mean of explained proportion (%)	SD	SEM
PI (*n* = 19)	PC1	68.40	15.79	3.62
	PC2	20.84	10.12	2.32
	PC3	10.76	6.60	1.51
HC (*n* = 21)	PC1	66.02	11.95	2.61
	PC2	23.31	8.44	1.84
	PC3	10.67	5.72	1.25

*PC principal component, *SD* standard deviation, *SEM* standard error of the mean.

### Performance of SVM classification

**Figure 5:**
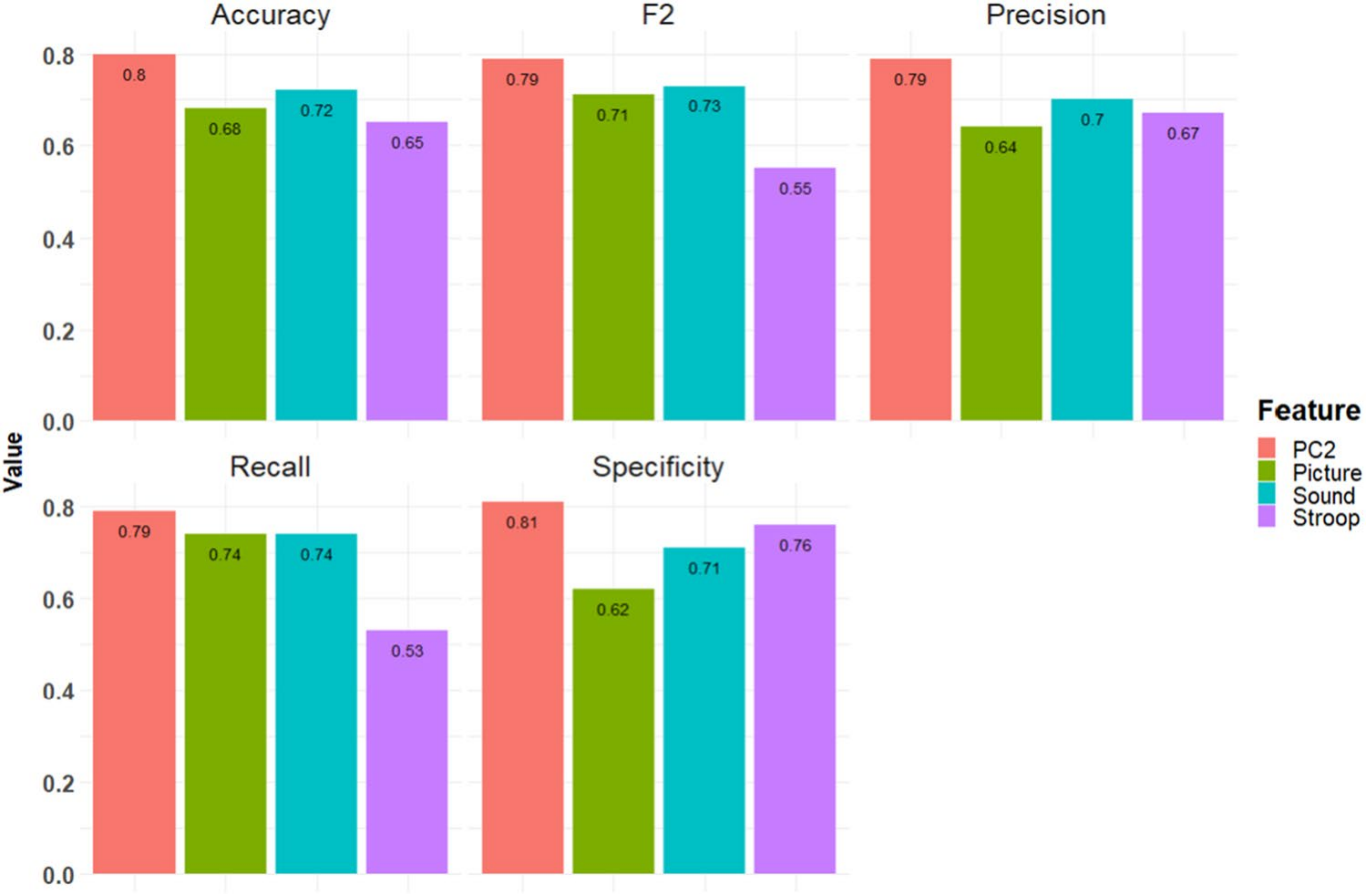
Results of performance metrics of SVM classification. The performance metrics resulted from SVM classification using input features of six PC2 loadings and six ROIbvals of picture, sound, and Stroop, respectively. The results showed that PC2 loadings achieved the highest scores for all performance metrics compared to ROIbvals of picture, Stroop, and sound. LOOCV was used as an evaluation of model fit.

To evaluate the prediction performance, we separated the data into patients with PI and HCs with four different feature sets selected from PC2, picture runs, sound runs, and Stroop runs; we evaluated five metrics as performance indexes: accuracy, F2, precision, recall, and specificity. As shown in Fig. 5, the respective accuracies of PC2, picture runs, sound runs, and Stroop runs were 0.80, 0.68, 0.72, and 0.65; respective F2 values were 0.79, 0.71, 0.73, and 0.55; respective precision values were 0.79, 0.64, 0.70, and 0.67; respective recall values were 0.79, 0.74, 0.74, and 0.53; and respective specificities were 0.81, 0.62, 0.71, and 0.76. Thus, performance metric analysis showed that the features selected from PC2 achieved the highest score in all performance metrics.

## Discussion

In the present study, in the context of task-induced functional connectivity (FC), we proposed a new approach for analysis of fMRI data using the integrating framework of multitask fMRI. We showed that the PC features generated from our method demonstrated better performance in distinguishing patients with PI from HCs, compared with the BOLD response features from individual fMRI stimuli. To create new PC features, we utilized three contrast images processed by SPM12 from three single-task fMRI scans of each participant, which were then decomposed into PCs based on the spatial covariance pattern of the contrast images by PCA. Finally, salient PC loadings were selected as input features by LASSO and validated by SVM.

We found that when comparing the PI and HC groups, the regions with different PC2 loadings combined with the response information for the three stimuli were the bilateral inferior frontal gyrus (orbital), right calcarine cortex (primary visual cortex), right lingual gyrus, left inferior occipital gyrus, and left inferior temporal gyrus. The findings suggest that these brain areas could distinguish the PI and HC groups on the basis of sleep-related stimuli. When we performed an activation likelihood estimation meta-analysis of task-based fMRI studies comparing HCs with patients with PI using seven task-related studies, only two clusters were identified (uncorrected *p* = 0.001, minimum volume = 400 mm^3^)^19,21,26–30^: left superior–medial frontal gyrus and left superior–middle temporal gyrus. There was a difference in comparison with the salient region derived in our study. One possible explanation for this difference is that a small number of articles were reviewed, and the cognitive tasks in each study were used mainly to evaluate frontal dysfunction (i.e., category fluency, letter fluency, and working memory). In addition, our results were not limited to a single stimulus or task because we combined multiple tasks (e.g., sleep-related visual stimuli, sound stimuli, and cognitive tasks). Moreover, there were differences among the study populations, because relatively homogenous patients with PI based on ICSD-2 diagnosis were enrolled in our study. Moreover, other sleep disorders (e.g., obstructive sleep apnea) were excluded via nocturnal polysomnographic and psychiatric evaluations in the current study.

The prefrontal cortex, including the inferior frontal gyrus, is a salient area identified by feature selection in patients with PI compared with HCs. This region contributes to a wide variety of executive functions, including attention, problem solving, and planning^38^. Reduced activation in the orbitofrontal cortex in patients with PI has been associated with cognitive disinhibition, such as rumination and intrusive thoughts^39^. During the performances of category and letter fluency tasks, patients with insomnia showed hypoactivation of the medial and inferior prefrontal cortical areas, compared with controls^26^. Similarly, Drummond et al. reported that patients with PI showed reduced activation of the right dorsolateral prefrontal cortex with increasing task difficulty^27^. Analysis of frontal lobe morphometry revealed gray matter reduction in the orbitofrontal cortex in patients with primary insomnia^40,41^. In an fMRI study with an amplitude of low frequency fluctuation algorithm, Li et al. reported that the duration of PI was negatively correlated with amplitude of low frequency fluctuation values in the left inferior frontal gyrus^42^. As such, PI is associated with structural and functional changes in the orbitofrontal cortex, compared with HCs, which are presumably involved in different responses to sleep-related stimuli.

Other areas with differences between patients with PI and HCs in PC2 loading included the inferior temporal gyrus, lingual gyrus, calcarine cortex, and inferior occipital gyrus. These are the areas of the brain responsible for “what you see” during visual processing. In addition, these regions are related to hyperarousal in patients with PI. Notably, patients with PI showed physiological hyperarousal compared with HCs, demonstrated by a greater maximum heart rate and spontaneous arousal index during polysomnography. Previous magnetic resonance imaging (MRI) studies have demonstrated functional and structural associations with sensory regions, including the visual cortex, in patients with PI. Killgore et al. reported that difficulty in falling asleep was associated with increased FC between the primary visual cortex and other sensory regions, such as the primary auditory cortex, olfactory cortex, and supplementary motor area, in healthy adults^43^. Moreover, in comparison with HCs, patients with PI tended to have increased cortical thickness in sensory regions, including the primary visual and primary auditory cortices^44^.

In our approach, we used PC loadings captured by PCA as input features for distinguishing patients with PI from HCs. Application of PCA to functional imaging data yielded new orthogonal PCs that captured the correlational structure among cortical areas^32^. Accordingly, PC loadings in our results can be interpreted as linear combinations of correlational weights of ROIbvals among cortical areas that respond to different domains consisting of picture, sound, and Stroop stimuli. For example, in the voxel-based PCA results, PC2 showed that sleep-related fMRI stimuli evoked functional correlation topology representing a strong positive correlation of the temporal cortex and thalamus, combined with strong negative correlation of the interior frontal cortex and lingual/occipital cortex. This functional network was important for distinguishing patients with PI from HCs. Recently, there has been a great deal of interest in testing group differences of brain FC in resting-state fMRI, which previously used mass univariate Pearson’s correlation or partial correlation matrices^45,46^. In a similar manner, our approach involved examining group differences in multivariate patterns of task-induced FC. With regard to capturing variation in original data, PC scores of Stroop picture and sound stimuli were 48.74, − 35.38, and − 5.08 in the PC1 axis (explained variation ~ 70%); 30.47, 43.95, and − 13.70 in the PC2 axis (explained variation ~ 20%); and − 6.72, − 4.86, and − 30.54 in the PC3 axis (explained variation ~ 10%), respectively (Supplementary Fig. S3). Based on the PC scores, PC1 appeared to be highly influenced by the BOLD response to picture and sound stimuli, but in opposite directions (picture/sound = 48.74/ − 35.38). This implied that PC1 captured an increased BOLD response pattern to picture stimuli and a reduced BOLD response pattern to sound stimuli in the same functional network, and that the inverse relationship was also true. As shown in Supplementary Fig. S2(a), the PC1 pattern represents an increased visuospatial area (parietal) and decreased auditory area (temporal). This pattern was reversed with respect to sound stimuli (e.g., increased auditory area). Similarly, the FC pattern of PC2 was highly influenced in the same direction by both picture and sound stimuli (picture/sound = 30.47/43.95). The FC pattern of PC3 was highly influenced by Stroop stimuli alone. Discrimination analysis revealed that the FC pattern of PC2 exhibited the highest performance among all input features. It is unclear why LASSO captured PC2. However, this result suggested that FC patterns shifted in the same direction by both picture and sound stimuli comprise an essential FC topology for distinguishing patients with PI from HCs.

To validate our approach, a nonlinear SVM was used, with features reduced by LASSO regularization. SVM is a commonly used machine learning method that has been widely used for pattern detection in neuroimaging, as it can effectively handle high-dimensional data and provide good classification results^47,48^. Our results showed that PC2 features generated by PCA consistently yielded better classification performance in all five metrics (i.e., accuracy, F2, precision, recall, and specificity), compared with features generated by BOLD responses to three single-task fMRI scans. Hence, these results demonstrated that our approach successfully investigated shared information through different domains of sleep-related stimuli. Single-task fMRI analysis has been widely used to identify specific cognitive processes related to our area of interest, neural involvement in psychiatric disorders^49^. These experiments were designed to understand the neural patterns specific to diseases, such as insomnia disorder. As explained above, PCA enables transformation of the original variables into new variables based on the correlation structure between the original variables. Our approach is helpful for understanding the underlying neural mechanisms of insomnia disorder by probing combined information among tasks of different functional domains. Another strength of this study was that all participants were tested by nocturnal polysomnography, which enabled exclusion of other sleep disorders. Furthermore, patients with PI were diagnosed with homogenous insomnia disorder after thorough psychiatric evaluation by clinicians.

Although large sample sizes are recommended in the fMRI field to increase statistical power and accuracy, this approach has not been widely implemented^50^. A primary reason is the considerable expense involved in collection of fMRI data and clinical information. Reflecting this real-world difficulty, the median estimated sample size for a single-group fMRI study in 2015 was 28.5 participants, whereas that for multiple-group studies was 19 participants (in our study, the numbers of patients and HCs were 19 and 21, respectively)^51^. Considering this practical point, our approach may offer a good alternative. In this study, we proposed multitask fMRI in a single participant and integrated useful information from each task fMRI by PCA. We also demonstrated better performance metrics for distinguishing patients with insomnia from HCs. This approach would be accessible method in real-world clinical field without much cost to cope with the lack of statistical power and poor accuracy in fMRI study.

The present study had some limitations. First, it used high-dimensional data (78 ROIbvals in the row direction) with a small number of tasks (three single-task stimuli in the column direction) for PCA. Under these conditions, the estimate of the leading PCs might have been inconsistent^36^. The PC axes can be determined by a few data points with high leverage. For example, the length and direction of the PC1 axis in our results were mainly determined by BOLD responses to picture and sound stimuli, which had high leverage. Therefore, the PC1 axis would be changed markedly by small changes in either picture or sound results. As shown in Supplementary Figure S4, for the PC1 axis, some cortical areas including superior/middle/inferior orbitofrontal cortex and rectus exhibited relatively large variances among participants. Therefore, estimates of PC loadings in these areas will be inconsistent. One solution may involve acquisition of a larger sample size (e.g., more single-task stimuli) within each participant, considering the limitations of the MRI equipment occupancy and the radiofrequency power delivered to the tissue. As the number of data points increases, the estimation of PCs may become more accurate, due to prevention of changes in the entire axis according to changes in single data points. Second, linear PCA analysis adopted in the present study works well in terms of linear characteristics among features. However, the pattern of spatiotemporal brain activity may produce higher than first-order spatial correlation (e.g., second-order spatial correlation), caused by interactions or modulations among brain areas^52,53^. Therefore, linear PCA analysis may not properly reflect complex non-linear feature spaces, such as those that may occur in neuroimaging data. A plausible method to address this issue was proposed by Tsatsishvili et al., who showed that nonlinear kernel PCA captured higher-order properties in fMRI experiments, which could not be captured by linear PCA^54^. Further studies involving nonlinear PCA are necessary to assess this limitation. Third, although we used relatively non-emotional sleep-related pictures, anything related to sleep might produce distress in patients with PI. In a future study, emotional and arousal valence levels should be controlled for more accurate assessment of between-group differences in attentional bias to sleep-related pictures. Fourth, resting-state fMRI (rsfMRI) was not included in the present study. Recent studies showed that rsfMRI had a similar sensitivity to that of task fMRI, as well as higher specificity in preoperative planning^55,56^. Thus, it may be advantageous to combine rsfMRI and task fMRI. In our previous research, we found significant differences in the resting-state FC of subcortical structures (e.g., basal ganglia, amygdala, hippocampus, and thalamus) with various cortical regions in patients with insomnia, compared with good sleepers^57^. These findings were insufficient to demonstrate an integrative explanation of the neural network in patients with insomnia, because the regions related to cognitive, emotional, and sensory arousal are interrelated. Thus, they have overlapping functions in the salience, somatosensory, motor, default mode network, and limbic networks. Other compensatory networks have not yet been revealed. Further studies are needed to evaluate whether a set of features from both rsfMRI and task fMRI yields higher classification accuracy compared with task fMRI.

In conclusion, our findings suggest that multitask fMRI using a machine learning approach has better performance in distinguishing patients with PI from HCs, compared with single-task fMRI methods. Additionally, the development of multitask fMRI data analyses might be useful to overcome the limitation of a small sample size in real-world research applications.

## Methods

### Participants

The participants consisted of 19 patients with PI and 21 HCs. Patients (aged 18–65 years) who met the ICSD-2 criteria for PI^5^ and HCs were both recruited from an outpatient clinic at the Department of Psychiatry and from the Center for Sleep and Chronobiology, at Seoul National University Hospital. Participants were excluded if they (1) had a past history of serious neurological or medical illness, (2) had current neurological or medical illness, (3) had other major psychiatric disorders including cognitive impairment or any personality disorder as defined by the DSM-IV^58^, (4) had sleep disorders other than PI, based on ICSD-2 criteria, (5) were shift workers, (6), had any contraindication to MRI, and (7) were pregnant women. The study protocol was approved by the Institutional Review Board of Seoul National University Hospital, in accordance with the Declaration of Helsinki (revised in 2008). Written informed consent was obtained prior to initiation of the study after all participants had been provided with a complete description of the study protocol.

To screen out psychiatric disorders, Structural and Clinical Interviews for DSM-IV axis I disorders were conducted in all participants by trained psychologists^59^. In addition, nocturnal polysomnography was performed to exclude individuals with common sleep disorders, such as obstructive sleep apnea, and physiological data (e.g., heart rate) were collected. Participants were instructed not to take any medications that could potentially influence sleep or circadian rhythm, such as sedatives, hypnotics, antidepressants, antipsychotics, or mood stabilizers. After a drug washout period of at least 5 days, those taking these medications may participate in the study.

### Clinical assessments

All participants completed several questionnaires, including the Pittsburgh Sleep Quality Index, Epworth Sleepiness Scale, Brief version of the Dysfunctional Beliefs and Attitudes about Sleep Scale-16, Beck Depression Inventory, and Beck Anxiety Inventory. The Pittsburgh Sleep Quality Index is a self-reported questionnaire that measures overall quality of sleep^60^. The Epworth Sleepiness Scale was used to assess daytime sleepiness; it is widely used to measure sleep propensity^61^. The Dysfunctional Beliefs and Attitudes about Sleep Scale-16 is a self-reported questionnaire regarding sleep-disruptive cognitions, such as faulty appraisals, unrealistic expectations, perceptual bias regarding sleep^62^. The Beck Depression Inventory and Beck Anxiety Inventory were used to measure severity of depressive symptoms and anxiety, respectively^63^. For continuous and categorical variables, the *t* test and chi-squared test were used, respectively, to examine differences in demographic and clinical measures between patients with PI and HCs.

### MRI data acquisition

A 3-T whole-body Siemens scanner (TrioTim Syngo; Siemens, Erlangen, Germany) with a 12-channel birdcage head coil was used for functional image acquisition with an interleaved T2*-weighted echo-planar imaging gradient echo sequence (repetition time = 3000 ms for picture stimulus, 2000 ms for sound and Stroop stimuli; echo time/flip angle = 30 ms/90°; slice thickness = 3.0 mm; in-plane resolution = 3.4 × 3.4 mm; field of view = 220 mm; matrix size = 64 × 64). The experiment was conducted in an fMRI room using an 8-in. screen; stimuli were presented by means of DMDX software. For each participant, the following numbers of functional volumes were acquired: 174 for picture runs, 185 for Stroop runs, and 159 for sound runs. After fMRI, an anatomical image was acquired using a high, T1-weighted, 3D gradient echo pulse sequenced with magnetization-prepared rapid gradient echo (repetition time/echo time/inversion time/flip angle = 1670 ms/1.89 ms/900 m/9°, slice thickness = 1.0 mm, in-plane resolution = 1 × 1 mm, field of view = 250 mm, matrix size = 256 × 256). The total duration of the experiment was approximately 40 min. Before MRI was performed, previous hypnotics or psychotropic medication were washed out for at least four half-lives of the drugs. The method of MRI data acquisition process is cited from Kim et al. study^21^.

### fMRI experimental procedure

The fMRI experiment consisted of picture, sound, and Stroop runs. Picture runs had a block design consisting of sleep and neutral trials, which were randomly intermixed across the run. Twenty-eight sleep-related and neutral pictures were selected from the Internet and used as sleep-related and neutral stimuli, respectively. Sleep-related pictures were validated by showing sleep-related images to 25 insomnia patients. Pictures were classified “sleep-related” if they were identified as “sleep-related” by more than 80% of patients with insomnia. The sleep-related pictures were designed to not trigger or contain any specific emotions in the general population. The neutral pictures were not related to sleep and also consisted of images that did not trigger any specific emotions. There were no emotional or facial expressions in either sleep-related or neutral pictures. For size and brightness, both types of images were matched. The method of validation process is cited from Kim et al. study^21^. In the stimulus epoch of each trial, four sleep-related or neutral stimuli were presented for 12 s. Within the epoch, each stimulus was presented for 3 s. After presentation of the stimulus epoch, a dot sign was shown for 2 s; the response sign was then presented for 2 s. Each trial was followed with a cross symbol for 12–20 s as a baseline. During the response sign, participants were asked to judge whether the stimuli were related to sleep. If stimuli were sleep-related, participants were instructed to press the right button with their right thumb; if stimuli were not sleep-related, participants were instructed to press the left button with their left thumb, following completion of the task. The sound run had a block design consisting of auditory sleep-related sound and non-sleep-related sound trials that were randomly intermixed across the run. A musical note symbol was presented on the screen during the sleep-related and non-sleep-related sound trials to signal the introduction of the sound to the participant. Four sleep-related sounds (two alarm sounds, one ticking clock sound, and one heartbeat sound) were sourced from the internet. The non-sleep-related sound was white noise. Sleep-related and non-sleep-related sound trials were each presented four times across the run for 20 s using magnetic resonance-compatible headphones. Each trial was followed by a silent period of 12–20 s as a baseline. The Stroop run had a block design consisting of incongruent and congruent trials that were randomly intermixed across the run. Incongruent items consisted of color-mismatched words (e.g., “red” displayed in blue), while congruent items were color-matched words (e.g., “red” displayed in red). An incongruent or congruent item was presented for 2 s within the epoch. Each trial consisted of 13 items. Each trial was followed by presentation of a cross symbol for 12–20 s as a baseline. During the trials, participants were asked to press one of four buttons as quickly as possible to indicate whether the word’s ink color was red, blue, green, or yellow, and the response time and accuracy were measured (Supplementary Fig. S5).

### fMRI analysis

fMRI data were analyzed using SPM12 (Wellcome Department of Cognitive Neurology, London, UK). The first six volume images were discarded from the analysis in all runs to eliminate the non-equilibrium effect. To correct for between-scan rigid body motion, the volume images were realigned to the first image in the time series. After realignment, the volume images were co-registered to the T1-weighted anatomical images and normalized to the Montreal Neurological Institute space using a transformation matrix derived from T1 anatomical image segmentation. After normalization, the volume images were spatially smoothed using an 8-mm full width at half maximum isotropic Gaussian kernel to compensate for residual between-participant variability after spatial normalization and to increase statistical sensitivity. The resulting fMRI time series was high-pass filtered using a cut-off time of 128 s to remove low-frequency drifts. A voxel-based general linear model was applied to each run to estimate the parameters, along with six motion parameters as covariates of no interest. In the picture run, two regressors were used to model the sleep-related and neutral stimuli. The response and instructional cue were also modeled to reduce the residuals. In the sound run, two regressors were also used to model the sleep-related and non-sleep-related sounds. In the Stroop run, two regressors were used to model the incongruent and congruent items. An instructional cue (i.e., a plus sign) was also modeled to reduce the residuals. A design matrix was applied to each run independently. Then, the design matrix was temporally convolved using a canonical hemodynamic response function to create a better fit. Finally, contrast images (beta values) associated with the conditions of interest (i.e., picture run: sleep-related stimuli—neutral stimuli; sound run: sleep-related sounds—non-sleep-related sounds; Stroop run: incongruent items—congruent items) were acquired for subsequent machine learning analysis. The above method description for fMRI data processing is cited from Kim et al. study^20^.

### PCA using multitask ROI beta values within participants

To perform ROI-based PCA, the ROIbvals were calculated based on the automated anatomical labeling (AAL) atlas (http://www.gin.cnrs.fr/en/tools/aal) using three contrast images (i.e., picture, sound, and Stroop), respectively, for each of the 19 patients with PI and the 21 HCs. For comparison, we used the AAL template. Since the AAL template was first implemented in SPM in 2002^64^, many fMRI results have reported activation areas based on the AAL template. Thus, we also reported our individual task fMRI results using the AAL template. Given the debate regarding the origin of the negative BOLD response^65,66^, voxels > 0.1*max in the ROI were initially selected before estimation of ROIbvals; this enabled consideration of positive beta values alone. After omission of barely overlapping AAL atlas ROIs among the participants, 78 of 90 ROIs in the cerebral cortex were finally selected. Areas not covered by the data were as follows: left olfactory cortex, right olfactory cortex, left amygdala, right amygdala, left superior parietal gyrus, right superior parietal gyrus, left paracentral lobule, right paracentral lobule, left temporal pole (middle), right temporal pole (middle), left pallidum, and right pallidum. Seventy-eight ROIbvals were transformed into matrix X with dimension p × *n*, where p is the number of ROIs (78 variables), and *n* is the number of contrast images (3 observations). After column-wise centering and scaling by $\sqrt{\text{n}-1}$, PCA was computed with a covariance matrix of matrix X using singular value decomposition:

1 $$ {\text{X}}_{{ij}}  = {\text{U}}_{{ij}}  \cdot \Sigma _{{ij}} ^{{1/2}}  \cdot V_{{ij}}^{T}  $$

where $\text{U}$ and $\text{V}$ are the matrices of eigenvectors of ${XX}^{T}$ (column space) and ${X}^{T}X$ (row space), respectively, while $\Sigma$ is a diagonal matrix of the eigenvalues $i=1, 2 \left(\text{ PI}=1,\text{ HC}=2\right), j=1, 2, \dots , {n}_{i}.$ In our study, the PCs represent the covariance structure between ROIs, and the eigenvalues represent the amount of variance explained by corresponding eigenvectors, compared with the original data set. After completion of PCA, three PCs were obtained from all participants. For further interpretation, PC images were obtained by voxel-based PCA using mean contrast images from patients with PI and HCs, respectively. PCA was performed using the “factoextra” package implemented in R statistical software (version 3.6.1; R Foundation for Statistical Computing, Vienna, Austria)^67^.

### Feature selection and classification using PC loadings

Following PCA analysis, the resulting PCs (${\text{U}}_{ij})$ were used as input features for construction of a classification model. Three PCs in ${\text{U}}_{ij}$ were ranked in descending order according to the proportion of explained variance. Initially, the first column of ${\text{U}}_{ij}$, PC1, was selected from each participant; a PC matrix with dimension p × m was constructed, where p is the number of ROIs (78 variables), and m is the number of participants (40 participants). This matrix used the following process of feature selection and classification; the process was also applied to the second column of ${\text{U}}_{ij}$, PC2, and the third column of ${\text{U}}_{ij}$, PC3. Feature selection using LASSO regression was performed to reduce the risk of overfitting and enable use of the proper number of features. LASSO limits the number of effective features via regularization of the L1 norm and allows selection of the most significant features (i.e., PC loadings) to construct the SVM classification. Logistic regression with LASSO finds fitting coefficients ${\widehat{\upbeta }}_{L}$, such that

2 $${\widehat{\beta }}_{L}= \underset{\beta }{\text{arg min}}\left[\sum_{i=1}^{N}\left({y}_{i}- \frac{1}{1 + exp(-\sum_{j=1}^{p}{\beta }_{j}{x}_{j})}\right)+ \lambda \sum_{j=1}^{p}\left|{\beta }_{j}\right|\right]$$

where $N$ is the number of samples, ${x}_{j}$ is the input feature, $p$ is the number of input features, ${y}_{i}$ is the outcome variable (i.e., $\text{PI }=1\text{ and HC}=0)$, and $\lambda$ is a regularization parameter of L1 norm. Overfitting of machine learning shows low bias but high variance, which caused poor reliability in the new data. We applied LASSO with L1 regularization parameter λ to avoid model overfitting. $\lambda$ limits model overfitting by influencing the degree of shrinkage of model parameters. Large $\lambda$ forces the small coefficient, ${\beta }_{j},$ to be zero, effectively resolving the small sample size problem. One hundred linear $\uplambda$ sequence values on the log scale ranging from ${\lambda }_{min}$ to ${\lambda }_{max}$ were searched, where ${\lambda }_{max}$ was set to a value at which all coefficients were zero, and ${\lambda }_{min}$ was set to a value at which the ratio of the smallest value to ${\lambda }_{max}$ was 0.01. The best regularization parameter, $\lambda ,$ was chosen as the value with minimum binomial deviance as a metric of model fit. Leave-one-out cross validation was used to evaluate model fit. After feature reduction using LASSO, the selected PC loadings served as input features for the classification model of nonlinear (radial kernel basis: $\mathit{exp}\left(-\gamma {\left|{x}_{i}-{x}_{j}\right|}^{2}\right)$) soft margin SVM.

3 $$\begin{aligned} & {\text{L}}\left(\widehat{\omega },\widehat{\text{b}}, \widehat{\xi }\right)= \underset{\omega ,b,\xi }{\text{arg min}}\frac{1}{2}{\Vert \omega \Vert }^{2}+C\sum_{i=1}^{n}{\xi }_{i}\\ & {\text{s}}.{\text{t}}., {y}_{i}\left({\omega }^{T}{x}_{i}+b\right) \ge 1- {\xi }_{i}, { \xi }_{i} \ge 0, i=1, 2, \dots , n\end{aligned}$$

where $w \text{and} b$ are hyperplane parameters, ${x}_{i}$ are input features (i.e., PC loadings selected in LASSO), ${y}_{i}$ is the outcome variable (i.e., $\text{PI }=1\text{ and HC}=0)$, and $\xi$ is the misclassification residual. $C$ is the cost of misclassification, and a greater $C$ value leads to greater variance and smaller bias. $\upgamma$ is the width of the Gaussian kernel, and a greater $\upgamma$ value leads to greater variance and smaller bias in the model. The SVM parameters $C$ and $\upgamma$ were optimized using grid search within a given range of $\text{C}$ sequence values, ranging from $0.1$ to $10$, and $\upgamma$ sequence values, ranging from $0.1$ to $10$. The best parameters, $C$ and $\upgamma$, were chosen as the values with the highest accuracy. Leave-one-out cross validation was used for evaluation of model fit. To determine whether the PC loading approach is a better predictor for classification of patients with PI versus HCs, compared with ROIbval, this process was also applied to ROIbvals of picture, sound, and Stroop runs. The logistic LASSO and SVM procedures were performed using the “glmnet” and “e1071” packages implemented in R statistical software (version 3.6.1; R Foundation for Statistical Computing)^68,69^. For classification performance metrics, five indices were used: accuracy, recall, precision, specificity, and F2. Each value was calculated as follows.

$$\text{Accuracy}= \frac{TP+TN}{TP+FP+TN+FN} , \text{Recall}= \frac{TP}{TP+FP} , \text{Precision}= \frac{TP}{TP+FP} ,$$

$$\text{Specificity}= \frac{TN}{TN+FN} , \text{F}2= 5*Precision\times Recall/(4\times Precision+Recall)$$

Accuracy is defined as the ratio of correctly predicted examples to the total examples, while recall (i.e., sensitivity) is defined as the ratio of correct positive predictions to the total positive examples. Precision (i.e., positive predictive value) is the ratio of correct positive predictions to the total positive predictions, while specificity is defined as the ratio of correct negative predictions to the total negative predictions. The F2 score is a metric that combines precision and recall, placing emphasis on recall.

## Supplementary Information

Supplementary Information 1.

## Data Availability

All data generated or analysed during this study are included in this published article (and its Supplementary Information files).
