# Supplementary material for: Multitask fMRI and machine learning approach improve prediction of differential brain activity pattern in patients with insomnia disorder
Source: Sci Rep. 2021 Apr 30;11:9402. doi: 10.1038/s41598-021-88845-w (PMC8087661; doi:10.1038/s41598-021-88845-w)
Supplement: Supplementary file 1 — Supplementary Information 1. [file 41598_2021_88845_MOESM1_ESM.pdf]

Multitask fMRI and machine learning approach improve prediction of differential brain activity pattern in patients with insomnia disorder

Mi Hyun Lee[1†], Nambeom Kim[2†], Jaeeun You[3], Hang-Keun Kim[3], Young-Don Son[3], Young-Bo Kim[4], Seong Min Oh[5], Soohyun Kim[6], Hayoung Lee[1], Jeong Eun Jeon[1], Yu Jin Lee[1*]

1 Department of Psychiatry and Center for Sleep and Chronobiology, Seoul National University College of Medicine, Seoul, Republic of Korea

2 Department of Biomedical Engineering Research Center, Gachon University, Incheon, Republic of Korea

3 Department of Biomedical Engineering, Gachon University, Incheon, Republic of Korea

4 Department of Neurosurgery, Gachon University Gil Hospital, Incheon, Republic of Korea

5 Department of Psychiatry, Dongguk University Hospital, Ilsan, Republic of Korea

6 Department of Neurology, Gangneung Asan Hospital, Gangneung, Republic of Korea

† Both authors contributed equally to this work.

* Correspondence and requests for materials should be addressed to Y.J.L. (email: ewpsyche@snu.ac.kr)

Supplementary Table 1. Selected features obtained by feature selection using LASSO

	PC2	Picture	Sound	Stroop
**Regularization parameter**	$\lambda = 0.0012$	$\lambda = 0.0189$	$\lambda = 0.0031$	$\lambda = 0.0064$
**Selected features**	Frontal_Inf_Orb_L<br>Frontal_Inf_Orb_R<br>Calcarine_R<br>Lingual_R<br>Occipital_Inf_L<br>Temporal_Inf_L	Frontal_Sup_Orb_R<br>Cingulum_Ant_R<br>Hippocampus_R<br>Calcarine_R<br>Lingual_R<br>Temporal_Pole_Sup_L	Supp_Motor_Area_R<br>Rectus_R<br>Cingulum_Post_L<br>Temporal_Pole_Sup_L<br>Temporal_Inf_L<br>Temporal_Inf_R	Frontal_Sup_Orb_R<br>Frontal_Inf_Orb_L<br>Frontal_Inf_Orb_R<br>Hippocampus_L<br>ParaHippocampal_L<br>Lingual_R

* Names of selected features were based on the AAL2 atlas. L = left, R = right, Frontal_Inf_Orb = inferior frontal gyrus (orbital), Frontal_Sup_Orb = superior frontal gyrus (orbital), Calcarine = calcarine cortex, Lingual = lingual gyrus, Temporal_Pole_Sup = temporal pole (superior), Temporal_Inf = inferior temporal gyrus, Cingulum_Ant = anterior cingulate gyrus, Cingulum_Post = posterior cingulate gyrus, Supp_Motor_Area = supplementary motor area, Rectus = rectus gyrus, ParaHippocampal = parahippocampal gyrus, Occipital_Inf = inferior occipital gyrus

Supplementary Figure

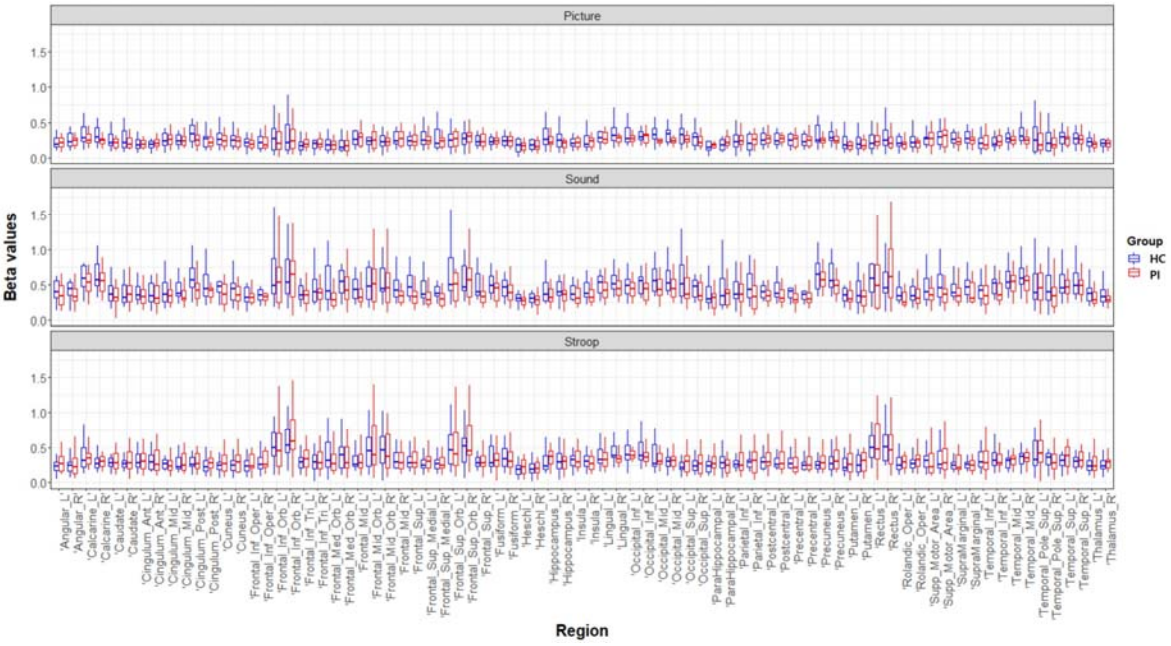

Supplementary Figure 1. Regional estimated median and range of ROIbval.

Regional distributions of ROIbval are shown as boxplots with horizontal line indicating the median, boxes indicating the interquartile range, whiskers indicating the maximum, and minimum for beta values (excluding outliers) for the PI (red color) and HC (blue color) groups. ROI names on the x-axis are based on the AAL2 atlas and stimulus types are indicated by rows.

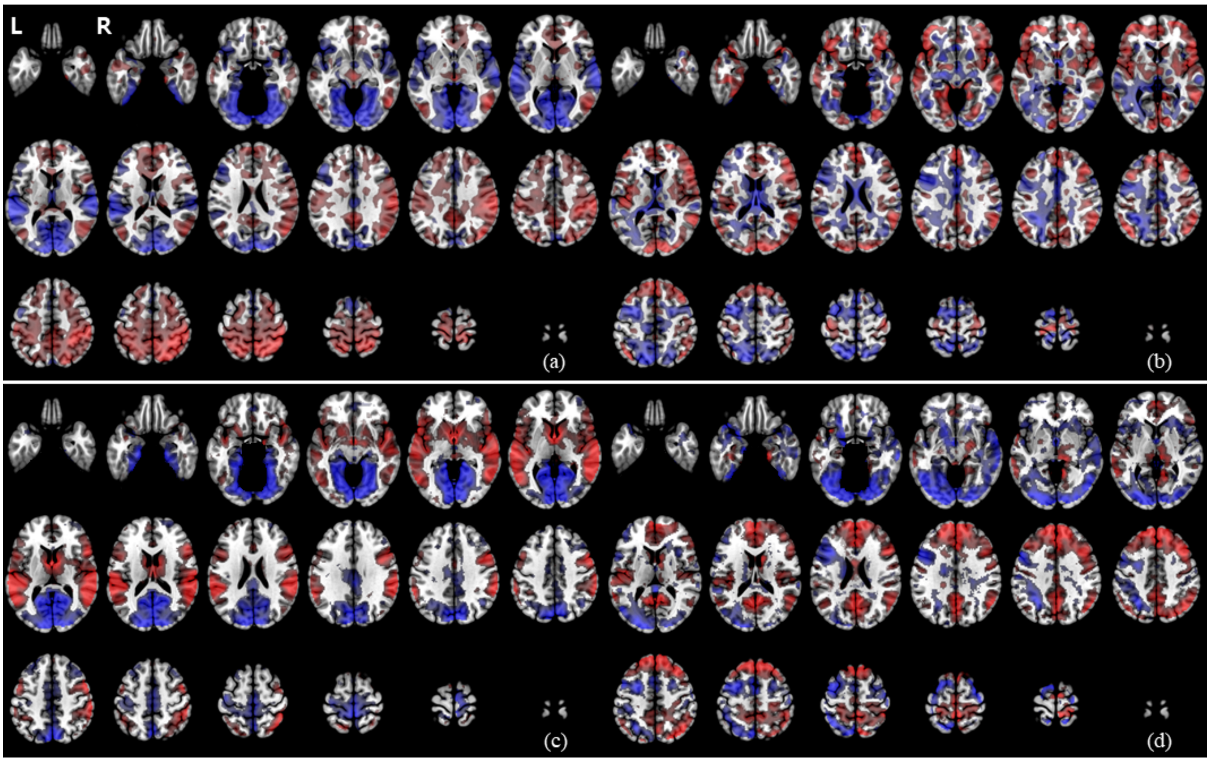

Supplementary Fig. 2 Representative PC1 and PC3 images of participants in the PI and HC group.

Voxel-based images were generated using PC loadings resulting from mean contrast images of picture, sound, and Stroop stimuli in the PI and HC group, respectively. Upper row showed PC1 (a) and PC3 (b) of PI and lower row PC1 (c) and PC3 (d) of HC. Red represents positive loadings and blue negative loadings indicating that neuronal activation was inversely correlated in these structures. The spatial patterns of PC1 and PC3 explained approximately 70% and 10% of the total variance of the raw data, respectively.

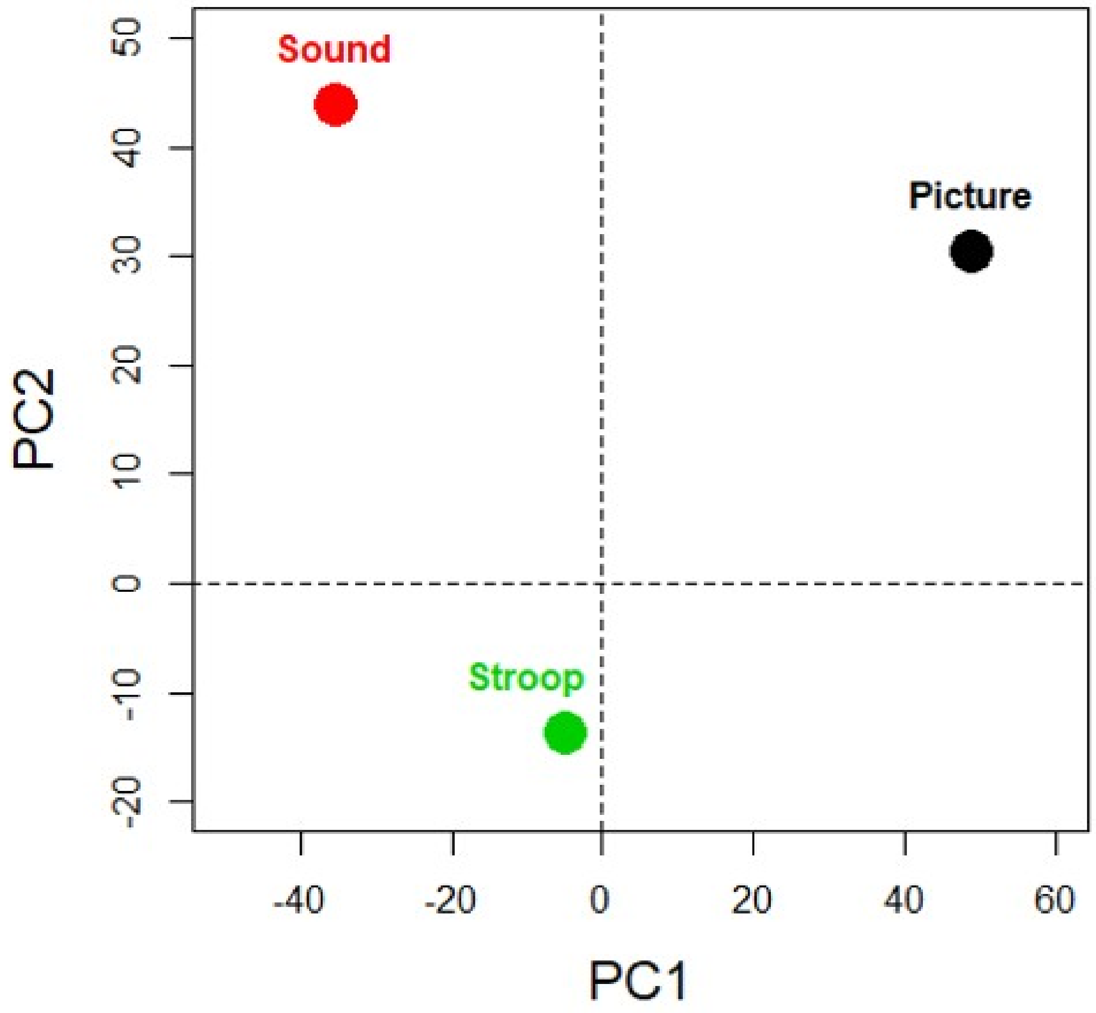

Supplementary Figure 3. PC scores of mean ROIbvals of picture, sound, and Stroop in insomnia.

PC scores were computed by projecting mean ROIbvals onto PC loadings (eigenvectors) of sample covariance matrix. This represents data characteristics that contribute most to data variation.

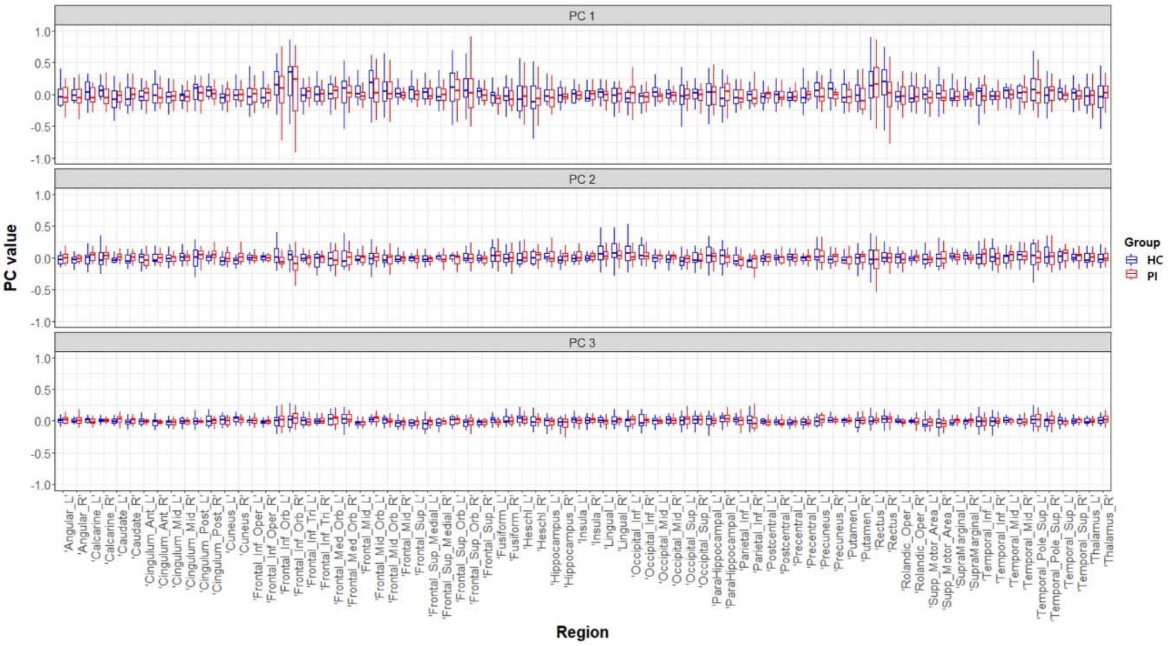

Supplementary Figure 4. Regional estimated medians and ranges of PC values.

Regional PC values distributions are shown for the PI (red) and HC (blue) groups, based on the AAL2 atlas. PCs are depicted by rows (excluding outliers).

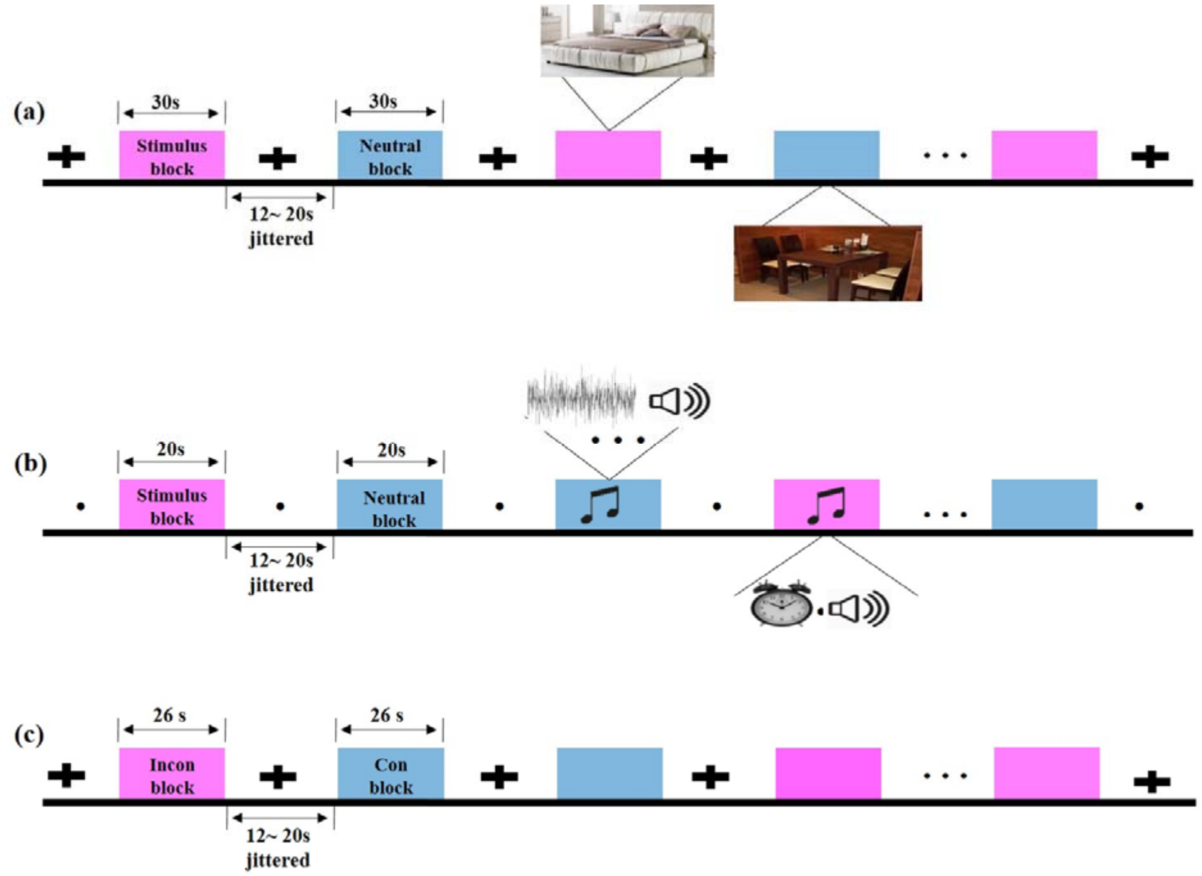

Supplementary Figure 5. Study design of the fMRI experiment.

The fMRI experiment consisted of picture (a), sound (b), and Stroop (c) runs within each participant. All runs had a block design consisting of target and control stimuli.[1]

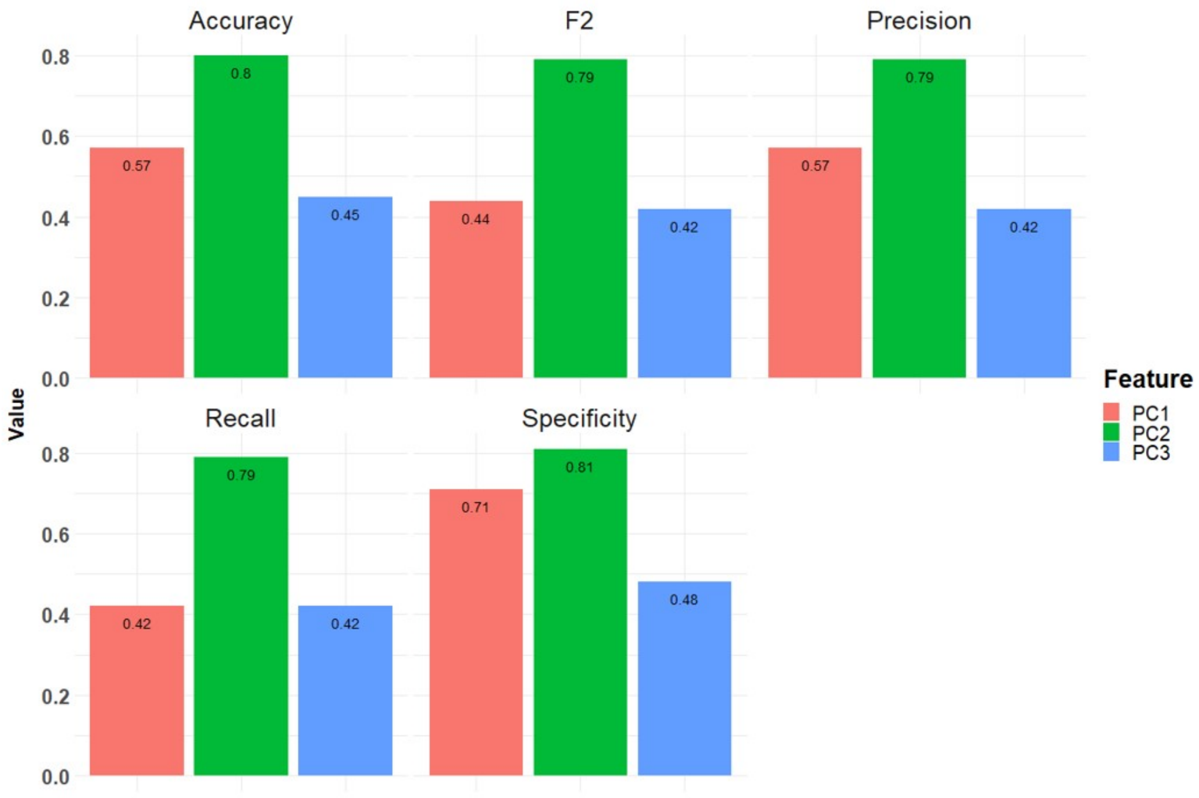

Supplementary Figure 6. Results of performance metrics of SVM classification on PC feature sets.

The performance metrics resulted from SVM classification using input features of six PC loadings of PC1, PC2, and PC3 selected by LASSO. The results of performance metrics showed that PC2 outperformed the other PCs in all performance metrics. LOOCV was used as an evaluation of model fit.

References :

1. Kim, N. *et al.* Decreased regional brain activity in response to sleep-related sounds after cognitive behavioral therapy for psychophysiological insomnia. *Psychiatry Clin Neurosci* **73**, 254-261, doi:10.1111/pcn.12822 (2019).
